# Printing‐Assisted Integration of Thermal, Fluidic and Electrical Components in Implants for Focal Brain Cooling

**DOI:** 10.1002/advs.77831

**Published:** 2026-09-27

**Authors:** Spencer Ryan Moore, Naomi King, Aruã Clayton Da Silva, Clare Howarth, Jason Berwick, Thomas E. Paterson, Ivan R. Minev

**Affiliations:** ^1^ Division of Polymer Biomaterials Science Leibniz Institute for Polymer Research Dresden Dresden Germany; ^2^ Else Kröner Fresenius Center for Digital Health Medical Faculty Carl Gustav Carus TUD Dresden University of Technology Dresden Germany; ^3^ School of Psychology University of Sheffield Sheffield UK; ^4^ Neuroscience Institute University of Sheffield Sheffield UK; ^5^ Department of Chemical Sciences SSPC the Research Ireland Centre for Pharmaceuticals Bernal Institute University of Limerick Limerick Ireland; ^6^ School of Clinical Dentistry University of Sheffield Sheffield UK; ^7^ Insigneo Institute University of Sheffield Sheffield UK

**Keywords:** 3d printing, electrocorticography, electronic component, electronics, fluidics, neuromodulation, neuroprosthetics, rapid prototyping

## Abstract

The limitations of electrical neuromodulation are driving the development of neuroprosthetic systems enhanced with complementary modalities. A key technological challenge however remains the integration of the necessary hardware components (optical, thermal, fluidic, electrical) into miniaturized implants. Here we present a design framework, inspired by fast prototyping, that promises to reduce the complexity, fabrication time, and device bulk of multimodal neural interface systems. By combining extrusion‐based 3D printing of silicones and discrete electronic components, we integrated thermal neuromodulation and electrical recording capabilities in soft packages suitable for placement on the cortical surface of rodents. When linked to an autonomous auxiliary driver unit, these packages establish rapid, stable and localized hypothermia together with ElectroCorticoGraphy (ECoG) recording. In a rodent model of epilepsy (4‐aminopyridine), we demonstrate hypothermia‐induced suppression of seizure‐like activity. Our approach may advance the clinical translation of focal hypothermia as an alternative therapy for drug‐resistant focal epilepsies.

## Graphical abstract

Extrusion‐based 3D printing of silicones and discrete electronic components are combined to create cortical implants for rodents that integrate thermal neuromodulation and electrical recording. Linked to an autonomous auxiliary driver unit, the system enables rapid, stable and localized hypothermia alongside ECoG recording. In a rodent epilepsy model, hypothermia suppresses seizure‐like activity.

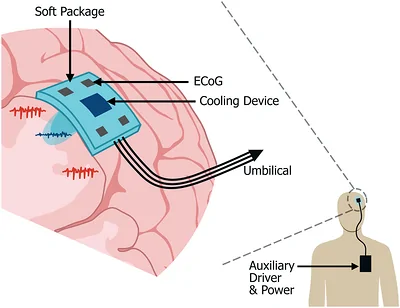

## 1 Introduction

Neuroprosthetic implants are designed to restore functions lost through injury or disease [1, 2, 3]. Key to this is the ability to modulate neural activity. The vast majority of established and emerging clinical systems couple electrical charges to the nervous system via Galvanic processes on electrodes [4]. Electrical neuromodulation however has limitations such as lack of specificity toward neuronal sub‐populations and circuits and encounters challenges with on‐demand inhibition of neural activity [5]. This has led to interest in neuromodulation via dosed and spatially defined delivery of optical (light for optogenetics), thermal (heating via pulsed infrared light), mechanical (focused ultrasound) or magnetic (transcranial magnetic stimulation) energy to the brain [6, 7, 8, 9]. In focal cooling, heat is extracted from a target region of the nervous system to temporarily suppress neural activity [10, 11]. It may be argued that one or more non‐electrical methods can be synergistically combined with electrical neural recordings. Such multimodal systems can enhance the specificity and selectivity of the interface, reduce stimulation artefacts in feedback signals, reduce side effects and provide a platform for exploring synergistic effects [12, 13].

**FIGURE 1:**
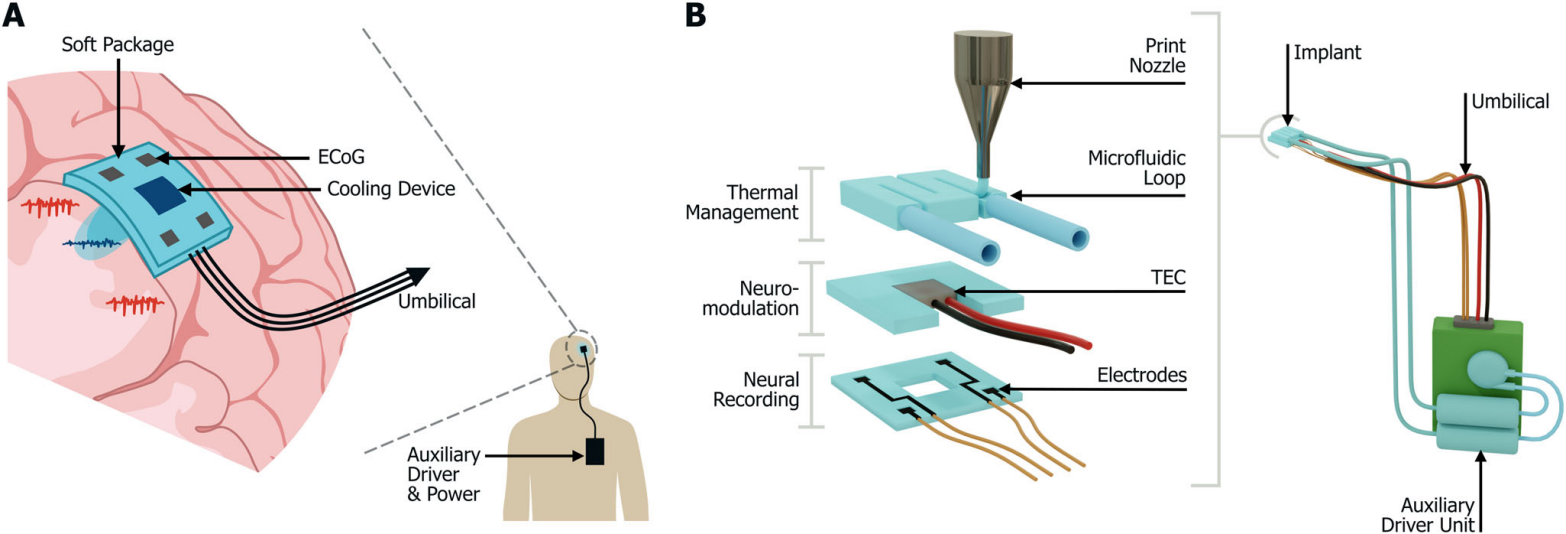
Modular design and printing‐enabled fabrication framework applied to focal brain cooling implants. (A) Concept of an epicortical implant for delivering focal thermal neuromodulation and neural recording. An auxiliary driver (ideally fully implanted) supports implant function and autonomy. Diagram designed in Inkscape (v1.4.2). (B) Modular design framework applied to the construction of the implant. It consists of three modules fabricated with the help of Direct Ink Writing (DIW) supporting neural signal acquisition, neuromodulation, and thermal management. The auxiliary driver unit supports the function of the individual modules through the umbilical connection. Render made with Blender 5.0 (Blender Foundation).

Despite pre‐clinical progress, there are currently few examples of multimodal neuroprostheses in the translational pipeline [14, 15, 16]. This gap likely stems from the difficulty of adapting existing biomechatronic design and materials frameworks to multimodal bioelectronics [17]. As an example, focal cooling systems promise a safe, reversible and non‐genetic suppression of neural activity, but have struggled to advance to clinical use for example in treating refractory epilepsy (Figure 1A) [18]. The deployment of components that can extract heat from localized areas of the brain is confounded by the accompanying need to safely transport and dissipate heat away from neural tissue. Implementation of thermal management by either passive or active means however has so far resulted in bulky and rigid hardware that is unsuitable for contact with soft brain tissue and implantation in the confined space of the skull [19, 20].

Multimodal implanted systems currently face a number of technological bottlenecks including: 1) integration of electrical, thermal, optical, and fluidic modalities, 2) miniaturization, 3) adaptation to the anatomical and spatial constraints of the nervous system and 4) incorporation of soft materials. The adaptation of printing technologies such as Direct Ink Writing (DIW), where viscoelastic inks are extruded through one or more nozzles moving along a computer controlled path, may address several of these challenges [21]. DIW supports integration of multiple functional materials such as conductors and insulators, and enables rapid, anatomy‐specific design iterations [22]. Although prior studies have reported printed implantable electrode arrays made from soft conductive composites and hydrogels, these devices are currently limited to electrical neuromodulation and recording [22, 23, 24].

Here we introduce a design and fabrication framework harnessing modular architecture, DIW and discrete electronic components to combine electrical, thermal and microfluidic functions in compact packages suitable for placement on the cortical surface of rodents. We linked implants to an auxiliary driver unit thus creating an integrated bioelectronic system. The system deploys, monitors and sustains cooling events without heating surrounding brain areas outside of the implant while simultaneously recording high fidelity ElectroCorticoGraphy (ECoG) from the surface of the brain. We validated our system using a terminally anesthetized rat model of epilepsy (4‐aminopyridine), demonstrating that focal cooling effectively reduces epileptiform activity. This proof‐of‐concept implementation advances the integration, autonomy, and customization of multimodal bioelectronic systems, bringing them closer to clinical translation.

## 2 Results

### 2.1 System Architecture

Our system comprises an epidural implant and an auxiliary driver unit. At the heart of the implant is a miniature (3.2×2.5×0.8 mm^3^) thermoelectric cooler (TEC), a solid‐state device using the Peltier effect to transfer heat from one side to the other upon application of an electric current. Although the temperature of the TEC's cold side (*T_c_*) can rapidly reach the range (15°C–30°C) required for hypothermia induced neuromodulation, on its own a TEC is not suitable for implantation. The heat transferred to its hot side can warm surrounding tissue beyond the permissible limit of 1°C above ambient brain temperature. Our modeling indicates that the TEC's hot side temperature (*T_h_*) cannot be maintained within safety limits by the natural flow of cerebrospinal fluid (Figure S1). Incorporation of heat pipes was deemed to add significant bulk and rigidity to the implant. To capitalize on the rapid and controllable cooling offered by TECs, we opted for a thermal management solution comprising of forced fluid flow and temperature monitoring. In addition to the components required for cooling, the implant incorporates an array of four electrodes for recording of the neuromodulation zone. Implants are built as stacks of modules where each supports thermal management, neuromodulation and ECoG recording (Figure 1B). The neural recording module is in contact with the brain surface and contains the electrodes, their interconnects and a large central through hole (via). The neuromodulation module contains the TEC, thermocouples, and a heat spreader bundled in a discrete package (Figure S2). The thermal management module is a microfluidics loop guiding fluid flow for heat removal. The implant package and the auxiliary driver are linked via a multimodal umbilical connection. The auxiliary driver powers and controls the functions of the implant modules via implementation of custom mechatronic hardware. It is responsible for supplying microfluidic flows, and current to the TEC, monitoring temperatures as well as ECoG signal conditioning and acquisition. The auxiliary driver has overall dimensions of 17×8×5 cm^3^ and can accommodate a battery (2S2P, Li‐Ion) for system portability.

### 2.2 Printing‐Assisted Implant Fabrication

**FIGURE 2:**
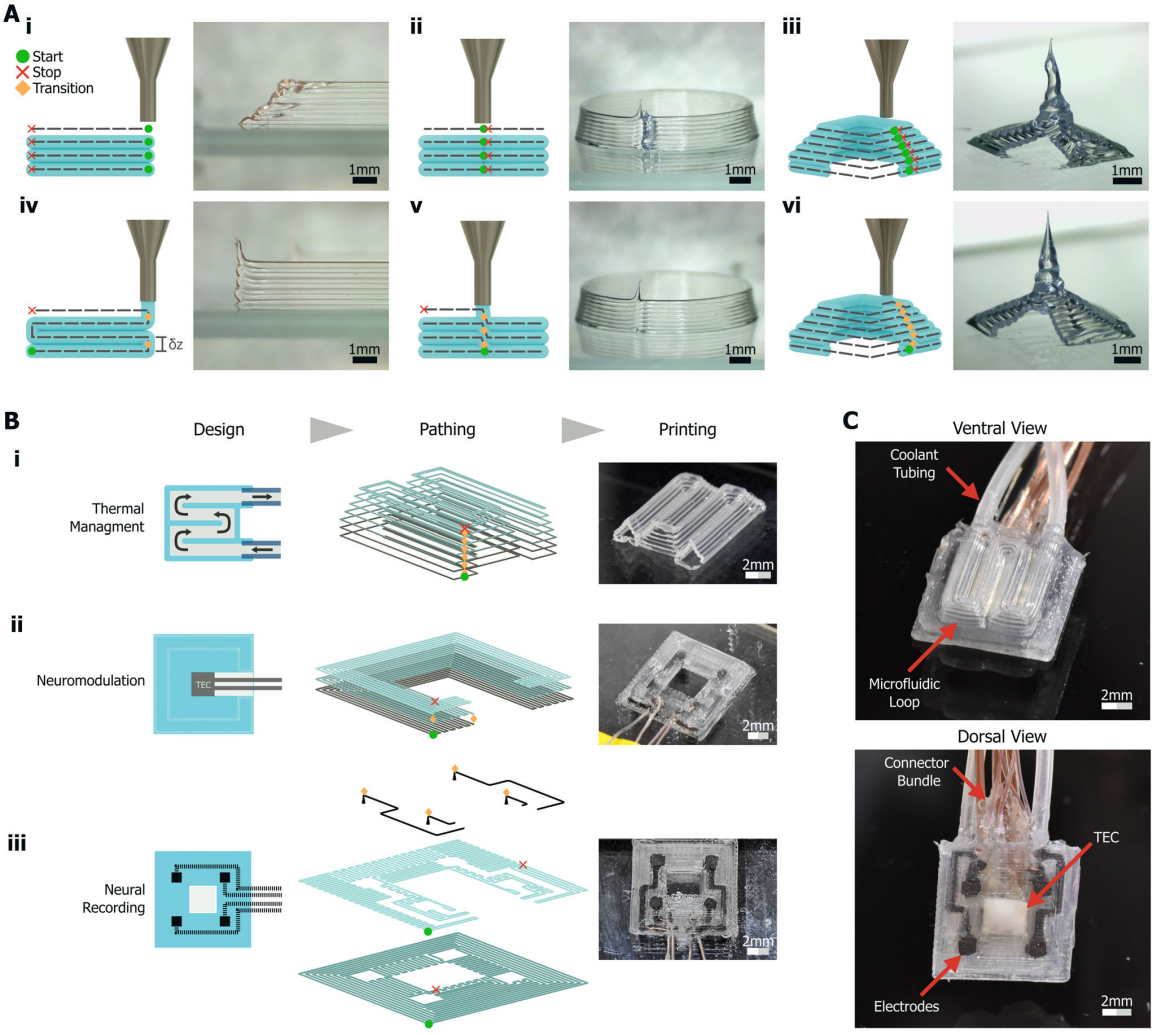
Implant assembly from printed functional modules. (A) Standard print paths with multiple start /stop points (i – iii) as compared to continuous print paths where start/stop points are replaced by smooth layer transitions (iv – vi) result in reduction of defects in freestanding silicone structures (wall, cylinder, hollow pyramid). Structures were printed using a nozzle with 0.234 mm internal diameter, print speed (printhead translation) of 5.5 mm s^−1^ and extrusion rate of 0.345 mm^3^ s^−1^. Diagrams (left panels) designed in Inkscape (v1.4.2). Right panels are optical micrographs. (B) Implementation of modules from concept to pathing design and printing. Continuous printing, combined with integration of discrete components and multi‐ink printing enables the realization of thermal (i), neuromodulation (ii) and neural recording (iii) modules. Diagrams (left panels) designed in Inkscape (v1.4.2), renders (middle panels) are designed in Blender 5.0 (Blender Foundation). Right panels are optical micrographs. (C) Integrated and connectorized implant package comprising the three stacked modules (optical micrographs).

The modules of the implant are fabricated by direct ink writing of a two‐component, shear thinning silicone (DOWSIL SE1700, Dow) that polymerizes into a soft and biocompatible elastomer through thermal curing. In a typical DIW process, the printhead follows an instructed path to fill consecutive 2D slices with extruded material to form a final 3D construct. This can result in defects caused by discontinuous print paths within slices and during z‐axis slice transitions (Figure 2Ai–iii). The resultant seam and interlayer defects can impact the dimensional accuracy, reproducibility and mechanical integrity of the modules. A significant improvement in print quality can be achieved by designing continuous print paths with uninterrupted material extrusion (Figure 2Aiv–vi). Once planned, continuous print paths can also reduce optimization efforts and improve the fidelity of self‐supporting structures that enclose a cavity. Otherwise, extra material that accumulates at seams can cause structure collapse (Figure 2Aiii, vi).

Continuous printing was applied in the path design of the implant modules. For the thermal management module, we realized a meandering (M‐shaped) microfluidic channel with a triangular cross section (Figure 2Bi). Channel input and output ports were exposed for attaching external flow connections. The neuromodulation module embeds the discrete cooling package within a printed structure tailored to its geometric and connectorisation requirements (Figure 2Bii). The neural recording module is printed from insulating (SE1700) and electrically conductive (graphite‐PDMS composite) silicones. Formed from a percolating network of graphite flakes dispersed in PDMS, the conductive composite is compatible with extrusion printing processes and our soft implant design framework [25]. Two layers of SE1700 were printed to create openings for the active sites of the electrodes and internal routing for the interconnects (Figure 2Biii). The conductive composite was deposited from a separate printhead. Layer transitions and printhead translation delays were adjusted to ensure consistent deposition of the conductive composite into the pre‐patterned electrode geometries. Starting from a non‐adhesive carrier substrate, each module is printed and partially cured to serve as substrate for building the next module (Figure S3). This stacking procedure ensures modules are electrically and thermally insulated whilst a final thermal cure results in a monolithic and defect free package (Figure 2C).

### 2.3 Auxiliary Driver and System Verification

**FIGURE 3:**
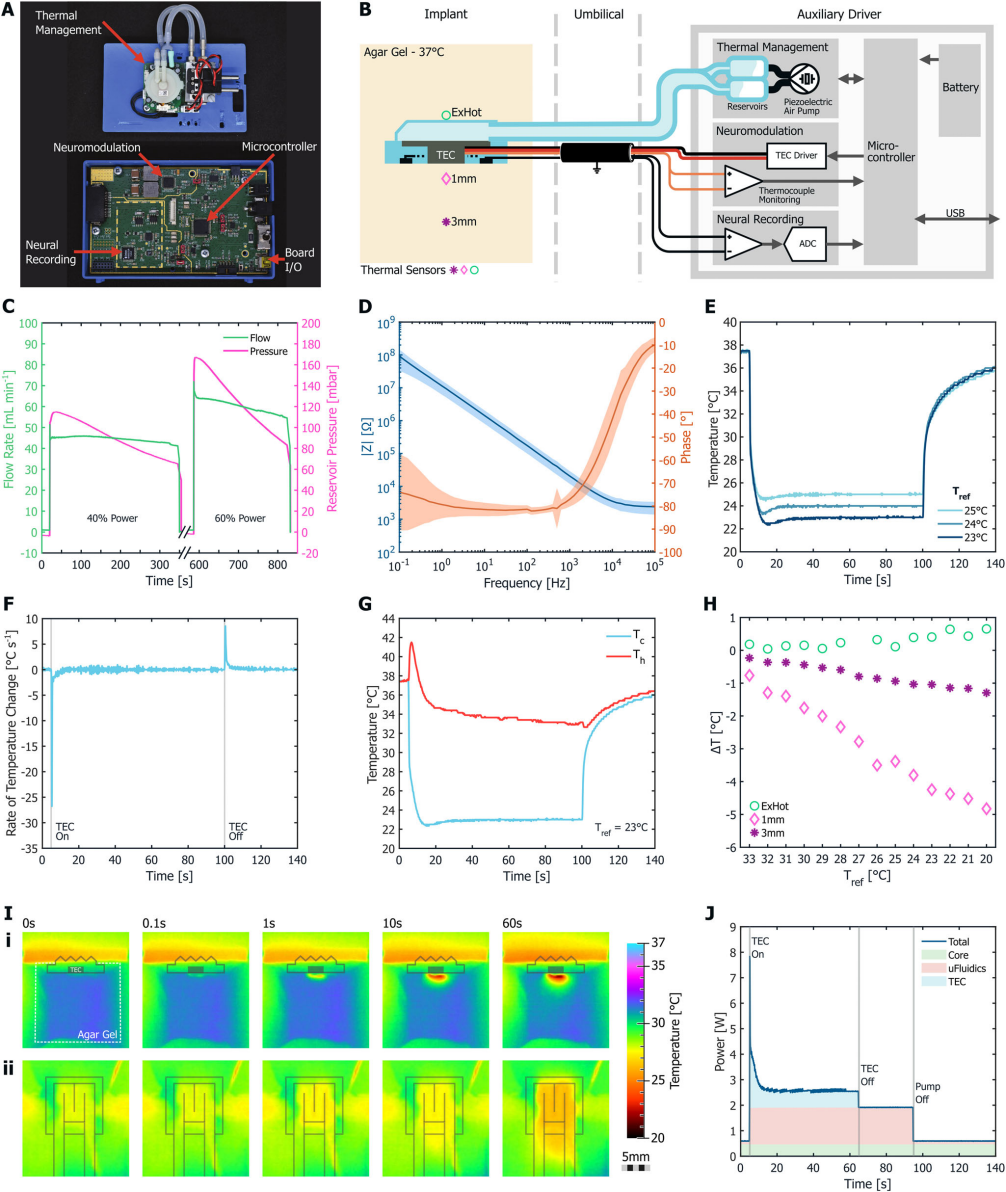
System integration and in vitro testing. (A) The auxiliary driver is a custom designed PCB hosting electronic components for each module as well as the piezoelectric air pump. The piezoelectric pump is used to alternately pressurize two interconnected water reservoirs (Figure S5) for supplying continuous water flow. (B) Overview of the system comprising of implant, umbilical connection and the auxiliary driver. Thermal sensors embedded in the Agar gel monitor the temperature at defined locations below and above the implant package. Diagram designed in Inkscape (v1.4.2). (C) Hydrodynamic characterization of the thermal management module. (D) Impedance modulus and phase angle of printed neural recording electrodes (1.2×1.2 mm^2^) obtained in Phosphate Buffered Saline (PBS). The averaged spectrum is indicated with solid line (± 1 S.D., shading). (E) Temperature profiles of the TEC cold side obtained over a set of *T_ref_* demonstrate stable control and maintenance of cooling. (F) Rate of *T_c_* change measured during establishment, maintenance and recovery from a cooling event (*T_ref_*  =  23°C). (G) Temperature profiles of the cold (*T_c_*) and hot sides (*T_h_*) of the TEC obtained during a cooling event (*T_ref_*  =  23°C). The deionised water in the thermal management module was maintained at 35°C, close to peripheral body temperature. (H) Spatial extent of implant driven cooling for a range of *T_ref_*. The Agar gel is maintained at an ambient temperature of 37°C to mimic the thermal environment of brain tissue. Here *ΔT* is the difference between temperatures measured by thermal sensors outside the implant and the ambient Agar temperature. (I) Thermal images of a side cross section (i) and top view (ii) of an implant delivering steady state cooling to *T_ref_*  =  15°C. (J) Contributions of key subsystems to the overall power consumed during a cooling event (*T_ref_*  =  22°C).

The components of the auxiliary driver are integrated on a custom printed circuit board (Figure 3A). At the center is a microcontroller (STM32U585, STMicroelectronics) managing the flow of incoming information and outgoing control signals (Figure S4). To enable rapid, controlled and stable cooling, we implemented a closed loop Proportional–Integral–Derivative (PID) controller where *T_c_* and *T_h_* are constantly monitored to adjust the current supplied to the TEC. To prevent thermal runaway of the TEC and controller instability, a piezoelectric air pump (UXPB5401200A, The Lee Company) was used to drive water circulation in the thermal management module (Figure S5). Neural signals are amplified, filtered and digitized by a discrete ASIC (RHD2216, Intan Technologies) linked to the microcontroller. A USB connection enables real‐time data transfer to a host PC for the purpose of data visualization and high‐level user programming.

Next, we verified the functionality of the implant modules coupled to the auxiliary driver (Figure 3B). The implant was embedded in a heated Agar gel to emulate the thermal environment of the brain. Thermal management was tested by setting up flow in the microfluidic system. Step power commands supplied to the pump resulted in predictable and sustained pressure and water flow in the microfluidic system (Figure 3C). Flow rates of at least 60 mL min^−1^ (reservoir pressure approx. 180 mbar) can be sustained without leaks in the printed microfluids section. Electrodes integrated in the neural recording module exhibit an average impedance of 20.4 ± 9.0 kΩ (at 1 kHz), measured from 16 electrodes of 5 implants. (Figure 3D). The fabrication process has a yield of 80% with exclusion criteria defined as electrodes having impedance above 50 kΩ. Under closed‐loop control, the TEC maintains accurate and stable cooling. We define *T_ref_* as the user‐programmed (i.e. the target steady‐state) temperature of the cold side of the TEC. In steady state, *T_c_* should be as close as possible to *T_ref_*. Over a range of *T_ref_*, the deviation of *T_c_* from the target was less than ± 0.25°C. When applied to a typical scenario (*T_ref_*  =  23°C), the reference temperature can be reached in approximately 5 s at an average cooling rate of 2.7°C s^−1^ (Figure 3E). By optimizing the PID control gains, a maximum instantaneous cooling rate of 27°C s^−1^ was attained by the neuromodulation and thermal management modules working together (Figure 3F). At activation, the hot side of the TEC (*T_h_*) experiences a transient heat spike, lasting approximately 10 s. This is overcome by the action of the thermal management module returning *T_h_* within the acceptable range (Figure 3G). Temperature sensors placed external to the implant confirm that the transient heat spike is confined to within the implant. In contrast, the cooling effect is dependent on *T_ref_* and is localized to a depth of 1–3 mm below the cooling surface of the implant (Figure 3H). To verify the spread of heat fluxes, we employed thermal imaging of the implant and the surrounding Agar gel. Thermal images corroborate the temperature measurements from thermal sensors in the Agar gel and suggest that laterally, the cooling effect is confined to tissue directly below the active cooling site (Figure 3Ii and Figure S6). We observe no parasitic heating due to wires supplying power to the TEC and that excess heat is not transferred outside of the thermal management module (Figure 3Iii). It should be noted that the Agar gel does not model the thermal effects of active blood and cerebrospinal fluid perfusion, thus likely overestimates the spatial extent of cooling (Figure S7). The power consumed by the system peaks during activation of the neuromodulation and thermal management modules and reaches a steady state of 2.6 W (*T_ref_*  =  22°C). During cooling activation, the TEC draws the largest share of the system power. However, in the steady state, and for a period following TEC deactivation, the thermal management module becomes the dominant power consumer (Figure 3J).

### 2.4 Cooling of Drug‐Evoked Seizures In Vivo

**FIGURE 4:**
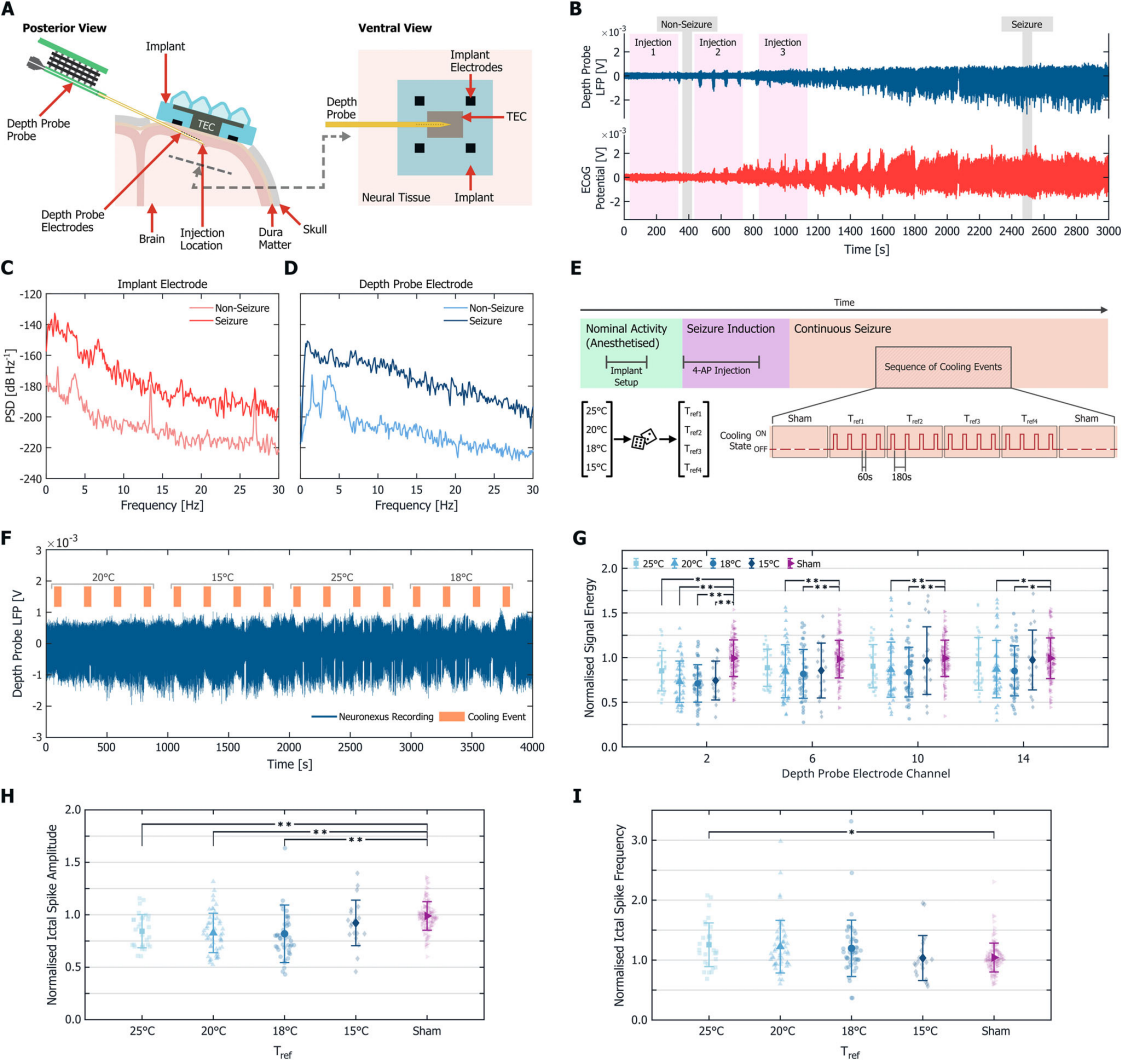
System validation in vivo. (A) Coronal section schematic illustrating the location of the epidural implant and the depth probe. The cortical surface is exposed via a cranial window. Inset on the right is a ventral view schematic of the depth probe and implant approximately illustrating their relative positions and sizes. Diagrams designed in Inkscape (v1.4.2). (B) Example of a parallel recording of Local Field Potentials (LFP) via the NeuroNexus depth probe and ElectroCorticoGraphy (ECoG) via the implant neural recording module. The signals are obtained during seizure induction. In this example, sustained seizure‐like activity emerges in approximately 40 min following three 4‐AP injections. Sustained seizures typically lasted several hours. (C) Example power spectrum density of ECoG (implant) recordings prior to seizure induction and during sustained seizure activity. Data is from 60 s sample windows similar to ‘Non‐Seizure’ and ‘Seizure’ periods shown in B. (D) Example power spectrum density of LFP (depth probe) recordings prior to seizure activity and during sustained seizure activity (60 s sample window). (E) Experimental protocol showing a sequence of cooling events. F. Timeline of a representative sequence of active cooling events and the corresponding LFP recording (obtained using E16, closest to the tip of the probe). (G) Normalised LFP signal energy. (H) Normalized ictal spike amplitude. (I) Normalized ictal spike rate. For G‐I, small symbols indicate the normalized measurable of the individual cooling events. The cohort average (large symbols) is obtained by first averaging within each experimental subject and then across the entire animal cohort. Error bars represent one standard deviation. Statistical significance is indicated with ^*^ when *p* < 0.05 and ^**^ when *p* < 0.01 (one‐way Kruskal Wallis ANOVA and Dunn‐Sidak Post Hoc processing).

The ability of the system to deploy rapid thermal neuromodulation was evaluated in a rodent model of epilepsy. The implant was placed epidurally on the sensorimotor cortex of urethane anaesthetized Hooded Lister rats (200–500 g). Gentle pressure was applied to ensure contact with the cortical surface (Figure 4A and Figure S8). Concomitantly, a depth probe (D16‐20mm‐100‐177, Neuronexus) was stereotactically inserted at a shallow angle, with the tip of the probe targeting a location 1–2 mm below the center of the TEC. The depth probe is equipped with a microfluidic channel for localized drug delivery from the tip, as well as 16 electrodes along the length of the probe shank (100 µm pitch, electrode E16 is closest to the tip). Model focal seizures were induced by injecting the potassium channel blocker 4‐Aminopyridine (4AP) via the depth probe. The depth probe records intracortical Local Field Potentials (LFPs) from individual electrodes referenced to the biological (animal) ground. In parallel, we performed differential recordings between diagonal pairs (spaced by 6 mm) of implant‐integrated electrodes. Thus, our ECoG recordings represent the LFP gradient at the surface of the cortex, selecting for neural activity originating in the cooled zone. Following 1–3 injections, 4AP establishes sustained seizure‐like activity observed in both depth probe LFP and implant‐integrated ECoG recordings (Figure 4B). During sustained seizure‐like activity, experimental animals maintained stable vital signs (Table S1), while both LFP and ECoG recordings showed a marked increase in power (relative to the anesthetized baseline) across a broad frequency range roughly spanning the delta, theta, alpha, and beta bands (Figure 4C,D) [26, 27].

Following the establishment of sustained seizure, we applied cooling events characterized by *T_ref_* (25°C, 20°C, 18°C or 15°C). Each cooling event lasted for 60 s after which the brain was allowed to rewarm for 180 s. The order in which the different *T_ref_* values were allocated was chosen at random for each experimental subject. A *T_ref_* was repeated four times sequentially before moving to the next *T_ref_*. Sham cooling events where the TEC was not switched on were conducted before and after a full set of active cooling events (Figure 4E). In rats where the seizure remained stable after a complete sequence of cooling events, a second sequence was undertaken. A representative sequence of active cooling events, alongside simultaneously recorded LFPs is shown in Figure 4F, illustrating a reduction of LFP amplitude within seconds of TEC activation. Cooling experiments were performed on 9 rats (Table S2). To evaluate the cohort‐wide effect of focal cooling on LFP activity, we calculated the signal energy during the last 30 s of each cooling event and normalized it to the signal energy of the 30 s preceding the cooling event (Figure S9). All tested *T_ref_* result in decrease of the average signal energy which may be interpreted as reduction in seizure activity. Although our data suggests that lower *T_ref_* results in greater suppression of neural activity, the deepest cooling events (*T_ref_*  =  15°C) resulted in a slight increase of the normalized signal energy suggesting the effect may not be linear [20]. We are however cautious to attribute a biological effect to this observation due to the limited number of trials at *T_ref_*  =  15°C (Table S2). The extent of LFP suppression does not appear to correlate with the position of recording electrodes on the depth probe. This is likely because all electrodes are located in a volume strongly affected by cooling (Figure S6). In addition, we used the LFP signal to extract the amplitude and rate of ictal spikes during cooling events (Figure 4H,I). We observe suppression of the amplitude of ictal spikes for all active cooling *T_ref_*, accompanied with a slight increase of their occurrence frequency that was significant only for the mildest cooling.

### 2.5 Integrated System for Deployment and Monitoring of Focal Brain Cooling

**FIGURE 5:**
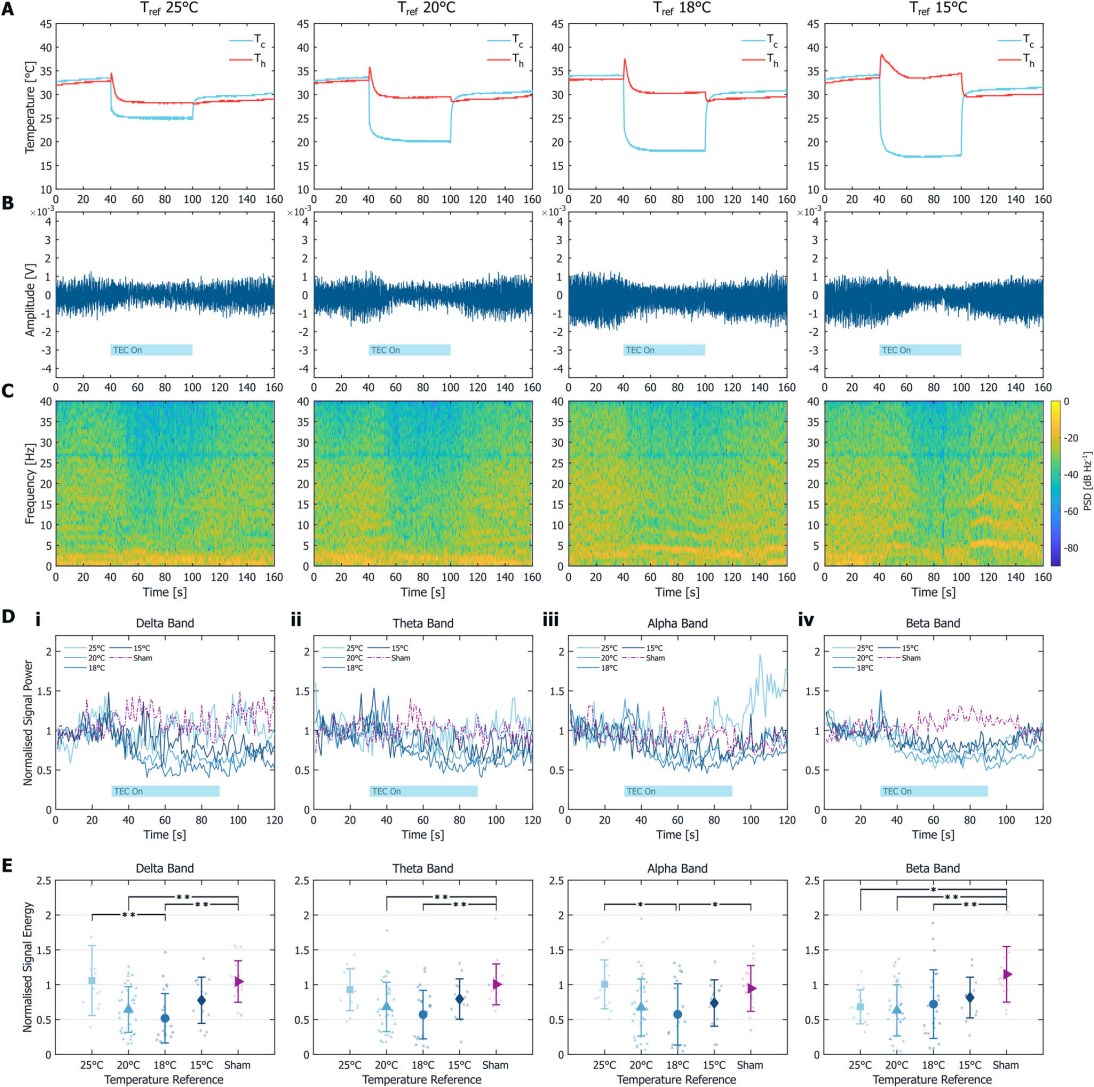
Integrated implant system. (A) Temperature profiles of the cold (*T_c_*) and hot sides (*T_h_*) of the TEC when the implant delivers cooling events of various *T_ref_* on the cortical surface. (B) Representative recordings from a pair of implant‐integrated electrodes illustrates the effect of cooling events on ECoG signal amplitude (*n*  =  1 rat). (C) Representative power spectrograms obtained from the ECoG recordings in B (1.5 s window, 0.5 s overlap, Hanning window). (D) Band power analysis using recordings from the entire experimental cohort (*n*  =  9 rats). Traces show the average normalised signal power (S.D. omitted for clarity) in each frequency band. Frequency bands are defined as (i) Delta (1–4 Hz), (ii) Theta (4–7 Hz), (iii) Alpha (8–12 Hz) and (iv) Beta (13–30 Hz). (E) Normalised signal energy in each frequency band and for each *T_ref_* (entire experimental cohort, *n*  =  9 rats). Error bars represent one standard deviation. Statistical significance is indicated with * when *p* < 0.05 and ^**^ when *p* < 0.01 (one‐way Kruskal Wallis ANOVA and Dunn‐Sidak Post Hoc processing).

We integrated the epidural implant with the auxiliary driver to demonstrate autonomous establishment and monitoring of focal brain cooling. The auxiliary driver is programmed to deliver a series of cooling events while simultaneously monitoring the thermal environment and brain activity (Figure 5A,B). The system is able to achieve similar cooling rates (approximately 2.9°C s^−1^) and *T_c_* stability (steady state variation less than ± 0.25°C) as observed during in vitro validation. Similarly, during cooling events, *T_h_* exhibited a brief thermal spike during the highest rate of cooling, before returning to a stable value during steady state cooling. Switching the TEC and microfluidic pump do not induce significant artefacts in the recorded signals (Figure S10). This demonstrates that the TEC, thermal management elements, control and recording algorithms are able to operate together with only abstracted user control (input of *T_ref_* sequence). As illustrated in Figure 5B, during sustained seizure activity, individual cooling events have a pronounced effect on ECoG signal amplitude which is in agreement with trends observed in depth LFP recordings. Cooling events appear to suppress neural activity in a broad band of frequencies above approximately 5 Hz (Figure 5C). Considering averaged data from the entire experimental cohort, the most consistent signal power suppression effects are observed in the delta and beta frequency bands (Figure 5D and Figure S11). Band power tends to return to pre‐cooling values several tens of seconds following the TEC deactivation, indicating that cooling events temporarily suppress but do not terminate the model seizure. For each frequency band, we calculated the ECoG signal energy during the last 30 s of each cooling event and normalized it to the signal energy of the 30 s preceding the cooling event (Figure 5E). Despite variability in the cohort as indicated by large standard deviation, significant energy reduction was detected in all frequency bands. For example, in the delta and beta band, a signal energy reduction of 48% ± 35% (*T_ref_*  =  18°C) and 31% ± 24% (*T_ref_*  =  25°C) respectively were calculated.

## 3 Discussion

Although there are currently no clinical neuroprosthetic systems that use focal cooling for treating drug‐resistant epilepsies, intraoperative demonstrations in humans have shown that mild focal hypothermia applied to the cortex delivers safe and effective neuromodulation [28, 29]. Recent work has advanced focal brain cooling hardware toward clinical translation, including multimodal probes that combine cooling with electrophysiological and hemodynamic monitoring and chronically implanted penetrating cooling probes demonstrating seizure suppression in non‐human primates [30, 31]. However, these systems still suffer from a low level of integration, with separate cooling and recording devices, bulky external or rigid backends (e.g., thermal baths, external fluidic loops), and the use of rigid metallic materials (titanium, stainless steel, copper) that limit miniaturisation and chronic implantability. Closed‐loop temperature control and fully implantable form factors remain largely unaddressed. Enabled largely by DIW rapid prototyping, our design framework addresses key challenges in system integration, the use of soft and biocompatible materials, and the implementation of multimodal actuation and sensing.

We demonstrated that implants for delivery of focal hypothermia can be fabricated using accessible 3D printing technology. This was achieved through careful design of print paths, selection of silicone inks and electronic components. Our implant prototype handles fluid flows (≈ 60 mL min^−1^), electrical currents (≈ 0.9 A), temperature differentials (up to ≈ 20°C), and millivolt neural signals in a compact (10×10×3 mm^3^) and mechanically soft package suitable for interfacing the cortical surface.

The functionality of our implant was validated in a rodent model, however its form factor and dimensions have more in common with human‐sized neuroprosthetic technologies. For example, cortical strip electrode arrays, used for seizure localization or monitoring in patients, typically support 4 to 12 recording contacts with diameters in the range of 2–4 mm embedded in a polymeric strip [32, 33]. Our printed device supports 5 contacts, where 4 are dedicated to ECoG (1.2×1.2 mm^2^) and one for focal cooling (3.2×2.5 mm^2^). Our implant adopts a planar form factor and delivers mild focal hypothermia to tissue at depths of 1–3 mm. This is comparable to the thickness of the human neocortex, making the design attractive for targeting focal neocortical seizures [10]. Distributed epileptogenic foci, foci buried in cortical sulci or those localized in deeper structures such as the hippocampus may require addition of more (distributed) focal cooling contacts, or redesign of the implant form factor [34, 35]. Future design adaptations are well‐suited to our modular, DIW‐enabled approach to implant fabrication.

The implant package is linked to support electronics and power via a multimodal umbilical link. We mounted all fluidic, electro‐mechanical and integrated circuit components (excluding the battery) on a printed circuit board occupying an overall volume of 680 cm^3^. Our auxiliary driver solution is far smaller than in previously reported focal cooling systems that rely on a back‐end of standalone pumps and rack‐mounted electronics [11, 30]. The auxiliary driver provides closed‐loop thermal control, autonomously executing user‐defined cooling events while preventing overshoot, oscillations, and off‐target heating outside of the implant. We achieved unprecedented cooling rates of nearly 3°C s^−1^ which is possible only with precisely controlled, thermally managed TECs. Rapid cooling is an advantageous system feature due to evidence suggesting faster onset allows suppression of epileptiform activity at milder hypothermic set points [36].

Our system is a bi‐modal neural interface where neuromodulation (thermal) and recording (electrical) are effectively decoupled. Unlike systems relying only on the electrical modality, this allowed us to monitor the effects of neuromodulation without interference from electrical stimulation artefacts [37]. The 4‐AP model allowed us to apply multiple cooling events (up to 20 in some of the rat subjects) to modulate a sustained baseline of seizure‐like activity. We observe that focal cooling results in reversible suppression of neural activity. Analysis of ECoG suggests that the extent of signal power suppression may weakly depend on the depth of cooling for milder hypothermias (*T_ref_*  = 25°C–18°C) but cooling becomes less effective when *T_ref_* is further decreased to 15°C. Why *T_ref_*  = 15°C is less effective than more moderate cooling remains unclear. As this result appears to conflict with previous studies showing that deeper cooling produces progressively greater seizure suppression, further experiments are needed to exclude technical factors or insufficient statistical power [36]. It is also important to note that the effects of cooling are likely non‐linear and may depend on complex neurovascular dynamics [20, 30]. Assessing additional seizure metrics, including seizure frequency, duration, and the proportion of time spent in ictal vs. non‐ictal states, may help identify the optimal temperature parameters for seizure suppression. Our analysis of LFP recorded by the depth probe reveals that the amplitude of ictal events is suppressed by cooling, but their rate remains less affected, even showing a slight increase at the mildest temperature. The reduced amplitude of LFP recordings in response to focal cooling is in line with previous cooling studies for both normal physiology and focal epilepsy [20, 38]. The small increase in rate frequency at 25°C can be explained by a biophysical transition where local network desynchronisation collapses the LFP magnitude, while altered ion channel kinetics can temporally elevate local discharge frequency [39].

Despite variations in ECoG recordings, cooling induced power suppression in the delta and beta bands showed statistical significance at the cohort level. This indicates that ECoG derived markers may be suitable feedback control signals to implement adaptive neuromodulation in an evolution of our system architecture [37, 40]. Future development may draw on control strategies from adaptive deep brain stimulation for Parkinson's disease, where continuous monitoring of beta‐band power guides stimulation intensity [41]. A closed‐loop implementation could combine a fast, threshold‐triggered activation of cooling at seizure onset followed by proportional control to titrate cooling depth during sustained seizures.

Monitoring of *T_c_* and *T_h_* during the in vivo experiments (Figure 5A) showed that steady‐state operation was reproducibly achieved within the duration of cooling events. This suggests that their duration can be readily extended beyond the 60 s used in this work, provided the PID control coefficients are adjusted accordingly. If required, 60 s cooling events could be applied in sequence, separated by brief rewarming intervals. Such an approach may be advantageous given that the effects of sustained cooling on brain tissue are not fully understood, particularly with respect to changes in local perfusion and metabolic inhibition [30]. The principal limit on continuous cooling duration is likely to be the power demands of the system which in its current iteration requires nearly 3 W during steady state cooling. For example, cooling for 10 min continuously would require the entire capacity of a small rechargeable battery (approximately 5 g, 140 mAh, 3.4 V) [42].

Several limitations of the current system will need to be addressed to advance its translation to the clinic. The auxiliary driver unit requires substantial further miniaturisation and integration to approach the dimensions of chest‐seated cardiac pacemakers and neurostimulators, which typically occupy volumes of 10–20 cm^3^. Achieving this will likely require the development of an application‐specific integrated circuit (ASIC) to reduce the PCB footprint, alongside miniaturisation and integration of the liquid reservoirs following design principles established for intrathecal drug delivery devices. A reliable and reversible connector will also need to be developed to link the auxiliary driver to the multimodal implant via the umbilical. This is analogous to the intraoperatively assembled connection linking electrode leads to the pulse generator in implanted neurostimulators. While power supply integration was outside the scope of this work, emerging wireless power transfer technologies currently being developed to support more energy‐intensive implants such as fully implantable mechanical hearts offer a path forward [43]. Finally, a hermetic titanium enclosure will be required to house the auxiliary driver and rechargeable battery for chronic implantation. The brain surface implant presents its own set of challenges. Its long‐term performance will need to be evaluated through accelerated ageing tests. In addition, the thermoelectric cooler is an active electronic component carrying substantial currents and may require supplementary packaging, potentially using liquid crystal polymer, to ensure reliable long‐term operation in the implanted environment [44].

We presented a design framework for rapid prototyping, fabrication, and integration of bioelectronic systems using non‐cleanroom tools and off‐the‐shelf components. This accessible workflow achieves form factors and feature sizes suitable for translational and clinical neuroscience. By lowering cost and improving flexibility, our approach can accelerate and democratize the development of multimodal implantable bioelectronics.

## 4 Methods

### 4.1 Preparation of Printing Inks

The two‐component printable silicone SE1700 (DOWSIL SE1700, Dow, USA) was prepared by mixing base and catalyst at 10:1 (wt:wt) ratio according to the manufacturer's instructions. The components are mixed and degassed in a planetary mixer (ARM‐310CE, Thinky Corporation) for 3 min at 2000 rpm. The conductive ink is a mixture of graphite powder and polydimethylsiloxane (PDMS). First PDMS (SYLGARD 184, Dow) is prepared using the manufacturer's recommended base to catalyst mixing ratio of 10:1 (wt:wt) ratio using the planetary mixer (3 min at 2000 rpm). Graphite powder (≤ 20 µm, 282863, Sigma Aldrich) is added to the PDMS pre‐polymer at a 1:1 (wt:wt) ratio. The composite is mixed by hand until the powder is incorporated in the PDMS pre‐polymer. Additional mixing in the planetary mixer is performed as described before until a homogenous mix is achieved.

The SE1700 ink is loaded into a clear 3 cc syringe barrel (QuantX, Fisnar, UK). A precision dispensing tip (I.D. 0.234 mm, micron‐s Fisnar, UK) is attached to the syringe barrel. The syringe is mounted onto the pneumatic stand dispensing (PSD) printhead of the printer platform (3D Discovery, RegenHU, Switzerland). The graphite‐PDMS conductive ink is loaded into a 2.5 mL Hamilton syringe. A precision dispensing tip (I.D. 0.335 mm, micron‐s, Fisnar, UK) is attached. The Hamilton syringe is mounted into the volumetric strand dispensing (VSD) printhead in the 3D Discovery. When not in use (including during partial curing steps), ink‐loaded syringes are stored in a fridge at 5°C which extends the ink lifetime to several days.

### 4.2 Path Design and Generation

Print paths were designed in the open‐source vector graphic software, Inkscape (v1.1.2). Each print layer using the SE1700 ink was designed to be one continuous path. Placement of the last node in a path is such that it aligns with the start node of the next path in the layer above. The completed paths are exported from Inkscape in the DXF12 file format and imported into BioCAD (v1.1, RegenHU), the proprietary G‐code generating software for the 3D Discovery. To generate G‐Code in BioCAD, the path material print settings are assigned; the paths print directions are corrected; and the paths are organised into the correct printing order. Material print settings are provided in Table S3 and the material assignment to the paths is shown in Table S4. Separate G‐code files are generated by BioCAD as follows – neural recording module (Layers 1 and 2); neural recording module (Layer 3); thermal neuromodulation module (Layers 4–6); and thermal management module (Layers 6–13). Each is considered a separate print due to the thermal curing required between print runs. To ensure continuous printing within each print run (can be one or more layers), the G‐code files are processed through a custom Python script. When designed, every path end node of one layer is aligned with the start node of the next layer's path. These discontinuous transitions are hand‐marked before feeding into the processing script. The script removes the inter‐layer G‐code and substitutes it with a G‐code command that makes the printer perform a z‐axis translation without halting ink deposition.

### 4.3 Preparation of the TEC Assembly

The package housing the TEC and thermocouples is assembled from discrete components (Figure S2). First 10 cm of PTFE insulated, copper wire (CZ1105, Cooner Wire, USA) is soldered to each of the TEC (1TC17‐16178‐1617.H, TECmicrosystems, Germany) terminals. To bond further elements to the TEC, a thermal‐cure epoxy (MED‐T7110, Epoxy Technology) is used. The epoxy is prepared according to the manufacturer's instructions and is dispensed from a syringe with a 0.510 mm blunt nose tip by hand. To monitor *T_c_*, a T‐type thermocouple (5TC‐TT‐TI‐40, OMEGA, USA) was positioned flat against the cold side of the TEC such that the sensor junction is in its the centre. A custom PDMS casting mould is mounted around the TEC to constrain the thermal epoxy to the dimensions of the cooling face. Thermal epoxy is poured into the mould until the thermocouple is encapsulated and epoxy is flush with the surface of the mould. This will form a ≈ 0.125 mm thick layer of thermal epoxy. The assembly is cured in the oven for 4 h at 60°C. To form a heat sink for the TEC hot side, a 6.3 × 6.3 mm square is cut from a sheet of stainless‐steel foil (0.2 mm Type 304, Fisher Scientific). The stainless‐steel square has a cross‐hatch pattern scored in its centre of one of its faces. It is mounted in a custom designed and 3D printed jig that aids in the alignment of the heat sink and the TEC during bonding. A ≈ 100 µm droplet of thermal epoxy is deposited on the scored area of the stainless‐steel square. The TEC hot face is aligned in the jig and pressure is applied with a clamp to hold the assembly in place. The setup is cured in the oven for 4 h at 60°C. To monitor *T_h_*, a thermocouple (same type as for *T_c_*) is placed to the right of the TEC hot side ensuring that the sensor junction is in contact with both the TEC hot side and the heat sink. The exposed thermocouple tip is potted with the thermal epoxy and cured in the oven for 4 h at 60°C. Using a brush, a silicone adhesive surface primer (MED6‐161, NuSil) is applied to the finished TEC assembly to ensure good adhesion to SE1700 during subsequent printing steps.

### 4.4 Implant Package Printing

A standard sized glass slide (75 × 26 × 1 mm) is used as the printing carrier substrate. The slide is cleaned with isopropanol, dried and sprayed with a release layer (Conformal‐Coating 422B surface modifier, MG Chemicals, Canada). This aids in the clean peeling of the implant from the glass slide after manufacture is complete. The prepared glass slide is mounted onto a custom slide holder with the coated side facing up and the holder is loaded onto the print plate of the 3D Discovery. This ensures the slide can be transferred to the curing oven and back to the same location on the printer plate. The printing process is summarised in Figure S3A.

The neural recording module is printed first (Figure S3B). The layers forming the electrode openings (Layer 1) and interconnect routings (Layer 2) are printed using SE1700 in a continuous extrusion run lasting approximately 2 min. The slide is removed from the printer and placed in an oven to cure for 40 min at 100°C. This partial cure prevents the printed silicone from wicking under the electrode faces, whilst also ensuring good adhesion of the subsequent printed layers. The slide is returned to the printer and electrodes are printed using VSD and the graphite‐PDMS ink (Layer 3). To link the printed interconnects, 36 AWG copper wires with PTFE coating are cut (10 cm), de‐insulated at the tips (1.5 mm) and manually inserted in the freshly printed graphite‐PDMS ink. A small amount of SE1700 is dispensed to secure the wires in place. The slide is then placed into the oven for 60 min at 100°C before being returned to the printer. Layers 4–6 are then continuously printed to bring the height of the sides to the same level as the TEC assembly and to create a guide for the assembly wires (Figure S3C). These layers are then cured in the oven for 60 min at 100°C. The thermal neuromodulation module is completed by placing the finished TEC assembly in the via formed in the neural recording module. A small amount of SE1700 is used to seal any small gaps. The slide is transferred the oven for 60 min at 100°C then returned to the printer plate. To form the microfluidic loop of the thermal management module, Layers 7–14 are realised using the SE1700 ink (Figure S3D). The slide is placed back into the oven for 60 min at 100°C. A scalpel is used to slice open the inlet and outlet of the cured thermal management loop. Two 10 cm long pieces of silicone tubing (I.D. 0.4 mm, O.D. 1.0 mm, Hilltop Products, UK) are cut. A ring of SE1700 is manually extruded onto one end of each tube piece. These ends are then inserted into the inlet and outlet ports on the microfluidic loop. Additional SE1700 is deposited around where the tubing enters the port to guarantee a water‐tight seal. A final curing step in the oven for 60 min at 100°C is performed. To enable the link to the auxiliary driver, the distal ends of the electrical wires are soldered onto a 12‐pin circular connector (micro360, Omnetics, USA). The soldered joints are over‐moulded with epoxy (MED‐T7110, Epoxy Technology) to ensure mechanical stability.

### 4.5 Auxiliary Driver

The auxiliary driver was designed using the KiCAD 6.0.9 EDA software and built in‐house from off‐the‐shelf components (Figure S4). Surface mount components were soldered to a custom 4‐layer PCB (Beta Layout GmbH, Germany) using the hot‐plate soldering method. Through‐hole components were hand‐soldered onto the PCB after. Neural signals from the printed electrodes were conditioned and converted to the digital domain using an off‐the‐shelf ASIC (RHD2116, Intan Technologies, USA). TEC control was enabled by a driver IC (DRV592, Texas Instruments, USA) with its power regulated by a buck‐boost regulator (MPM3650, Monolithic Power Systems, USA). The thermocouple readings were conditioned and converted to the digital domain by a thermocouple amplifier IC (MAX31855, Analog Devices, USA). Power to the neural recording interface and thermocouple amplifiers was regulated by a low‐noise, linear regulator IC (LT3045, Linear Technologies, USA). The thermal management system coolant (deionised water) is driven by a piezo‐electric air pump (UXPB5401200A, Lee Company, USA) mounted on a control module (SPM‐04, Lee Company, USA). Pump air flow direction is controlled using two 3‐way solenoid valves (18801105 Series‐188, Emerson, USA). Coolant fluid flow rate is monitored with a flow sensor (SLF3S‐1300B, Sensirion, Switzerland). Details of the pump subsystem of the auxiliary driver are presented in Figure S5. All subsystems of the auxiliary driver were coordinated using a microcontroller (STM32U585RI, STMicroelectronics, Switzerland). A power monitor (PAC1944, Microchip Technology, USA) is used to independently monitor the consumption of the whole auxiliary driver; the TEC unit; and the thermal management pump system. The microcontroller and system power monitor had its power regulated by a linear regulator (LT3039, Linear Technologies, USA) separate from the analogue supply. The 16‐pin Mini‐Dynamic connector system (TE Connectivity, USA) is used on the PCB to interface the axillary driver with the umbilical cable to the implant.

### 4.6 PID Controller

A closed‐loop control system is implemented for achieving and maintaining *T_ref_*. A proportional‐differential‐integral (PID) controller is implemented in the main programme loop of the microcontroller firmware. The controller is updated at a rate of 8 Hz to align with the thermocouple amplifiers conversion time boundaries and firmware data collection interrupt scheme. The base PID controller gains were set to *K_p_*  =  12 *K_i_*  =  0.98, and *K_d_*  =  0. Limiters were put in place throughout the PID controller to prevent the system temperatures exceeding the safe temperature range for neural tissue during operation. The temperature reference input of the controller was limited to the range 10°C–37°C. If an out‐of‐range value was provided, the temperature reference would default to the closest limit. An anti‐windup limiter was programmed onto the output of the integral term. This is to prevent integral over‐saturating during longer TEC operation and/or reaching lower temperature reference. The PID output is interpreted as a 0%–100% value of the maximum TEC driving current. This range was restricted such that there was an upper output limit of 90%. This value was derived during the testing of the heat throughput capabilities of the thermal management module. A safety shut‐off system was implemented to handle cases of TEC thermal runaway. In the event that the TEC hot‐side reaches above 45°C during a cooling event, the shut‐off system activates and the TEC driving current is reduced until the TEC is off. Cooling is then attempted up to two more times before the system cancels the commanded cooling event.

### 4.7 Graphical User Interface

High‐level control and monitoring of the system were achieved through a custom LabVIEW (2023 Q1) graphical user interface (GUI). Only the core LabVIEW package is utilised. The programme is structured in a producer‐consumer design to decouple data collection from programme processing lag. The GUI ingests the data stream outputted by the auxiliary driver to provide real‐time data visualisation. A number of charts were used to separately monitor TEC temperature, neural recordings, and TEC driver control data. A set of user commands were designed into the auxiliary driver firmware to provide control over the implant system. Virtual buttons and input fields on the GUI facilitate user interaction.

### 4.8 Electrode Characterization

Electrodes were characterized by Electrochemical Impedance Spectroscopy (EIS) using a potentiostat/galvanostat (PARSTAT3000, AMETEK) controlled by VersaStudio 2.60.2 software. The integrated implant was placed in phosphate buffer saline (PBS, pH = 7.4) and the individual electrodes were connected as working electrode. A Ag/AgCl/KCl 3 m reference electrode (BASi MF‐2056, BASi, USA) and a platinum wire coil counter electrode (BASi MW‐1033, BASi, USA) were used to complete the cell. EIS data were recorded from 1 MHz to 0.1 Hz, with an excitation amplitude of 10 mV (RMS) at 10 points per decade.

### 4.9 In Vitro Thermal Model

Aqueous Agar gel (1% wt.) was used as surrogate for neural tissue during in vitro thermal testing. At this concentration the gel is expected to have thermal conductivity of approximately 0.5 W m^−1^ K^−1^ which is similar to neural tissue (Tissue Properties Database V4‐0, IT'IS Foundation) [45]. The gel was produced by gradually adding Agar powder (05040, Sigma Aldrich) to boiling water under vigorous stirring until a homogeneous solution at the desired concentration was achieved. Heat was maintained for 5 min and then the solution was allowed to cool to 55°C before casting in a beaker. Before the gel set, the implant and thermal sensors were embedded. One thermal sensor was placed on the outer surface of the thermal management loop. Two further temperature sensors were placed at 1 and 3 mm below the cold face of the TEC. The beaker was transferred to an incubator (Challenger MX25, dip‐slides.com, UK) and maintained at 37°C. The coolant reservoirs of the auxiliary driver unit were also maintained at physiological temperature inside the incubator. During testing, 95 s of active cooling (TEC ON) was followed by rewarming (TEC OFF). The thermal management module (coolant circulation) remained active for 10 s after the TEC is switched off to prevent a thermal spike. Temperatures were continuously monitored by external and internal sensors.

Thermal images of the implant were captured using a thermal camera (U5855A, Keysight, USA) and analysed with the TrueIR Analysis and Reporting Tool software (v2.0.60920.01, Keysight, USA). The camera lens was placed 100 mm above the imaging target and manually focused to the target surface. To facilitate imaging the Agar gel was sliced to expose either the top of the thermal management loop or a cross‐section under the TEC cooling face. During imaging, the agar model with the embedded implant and coolant reservoirs were maintained at 37°C in the incubator. Cooling events were similar to those applied in vivo (60 s of active cooling, followed by 30 s continued thermal management module activity).

### 4.10 Rat Surgery

All animal procedures were performed in accordance with the guidelines and regulations of the UK under home office project licence number (PP0289372). The UK Home office project licence is issued under the Animals (scientific Procedures) Act 1986 (ASPA) which is fully equivalent to an IACUC approval. Every project license requires local ethical review by the Animal Welfare and Ethical Review Body (AWERB) before being submitted to the Home Office.

Both female (230–330 g) and male (230–500 g) Hooded Lister rats (RRID:RGD_2312466, sourced from Charles River Laboratories, UK) were separate sex housed in a temperature‐controlled environment (20°C–22°C) under a 12 h dark/light cycle with ad libitum access to food and water. Experiments were conducted under terminal urethane anaesthesia. Initial temporary sedation with Isoflurane (3% in 100% oxygen) was followed by intraperitoneal injection of urethane (1.25 g kg^−1^). Additional doses of urethane (0.25 mL, i.p.) were administered to ensure a surgical plane of anaesthesia, confirmed by absence of the pedal withdrawal reflex. Core temperature was maintained at approximately 37°C using a homeothermic heating blanket system equipped with a rectal temperature probe (Harvard Instruments, Edenbridge, UK). Cannulation of the femoral vessels was performed in a subset of animals (*n*  =  4) to confirm that key physiological parameters in blood remained within an acceptable range during the experiment. An i‐STAT 1 blood gas analyser using CG8 cartridges (Abbott, USA) was used to determine systemic pH, pCO_2_, oxygen saturation and others pre‐surgery, post craniotomy and at the end of cooling experiments (Table S1). Rat heads were secured in a stereotaxic frame (Kopf Instruments, California, USA) for the duration of the experiment. The scalp was removed to expose the skull. A square window with the dimensions of the implant (10×10 mm) was exposed using a dental drill. The window overlays the right somatosensory cortex (Figure S8B). The dura mater was kept intact. To minimise thermal injury, sterile saline was used for cooling during drilling. Any bleeding was managed with saline or cauterisation.

### 4.11 Positioning of Depth Probe and Implant

A dummy silicone implant (10×10 mm) was positioned in the craniotomy window. Instead of a TEC, the dummy implant had a hole (via), allowing us to visualise and mark the area. The dummy implant was removed. The depth probe (D16‐20mm‐100‐177, Neuronexus, USA) was mounted on a micromanipulator and inserted at a shallow angle from the direction of the midline such that it lay laterally to the surface of the brain. Care was taken to avoid major blood vessels. The depth probe was visible from the surface of the brain and was advanced until its tip reached the centre of the marked area (under the TEC).  A small amount of sterile saline was placed on the surface of the brain to prevent drying and to allow contact to the electrodes of the implant. A functioning implant was placed in the craniotomy window. To ensure good contact between implant and dura mater, gentle pressure was applied with the help of cotton swab (Q‐tip) attached to a micromanipulator. Contact was verified by inspecting ECoG signals in real time.

### 4.12 Seizure Induction

The drug infusion port of the depth probe was connected to a Hamilton syringe within a syringe pump using small‐bore silicone tubing. The system was pre‐loaded with 15 mmol 4‐aminopyridine (SLS, UK). The drug was delivered in doses of 1 µl at a rate of 200 nL min^−1^. ECoG signals were monitored to evaluate the emergence of seizure‐like activity. Establishment of sustained seizure‐like activity required the administration of up to 3 doses of 4‐AP. Sustained seizure like‐activity is deemed to be established when a continuous train of ictal events is observed.

### 4.13 Cooling Protocol

Four system temperature references, *T_ref_* (15°C, 18°C, 20°C, and 25°C) were selected as targets for all active in vivo cooling events. The order at which *T_ref_* are applied within a cooling sequence was randomised at the beginning of each rat surgery. Each *T_ref_* was applied four times sequentially, before applying the next *T_ref_*. Active cooling had a duration of 60 s (TEC ON). The thermal management module is activated together with the TEC and operates for a further 30 s after TEC OFF to ensure heat on the TEC hot side is safely dissipated. An interval of 180 s was maintained between each cooling event to allow the seizure to return to baseline activity. Time slots before and after the sequence of *T_ref_* cooling events, where the TEC and thermal management module were dormant, was allocated to sham cooling events. In rats where the seizure remained stable after a complete sequence of cooling events, a second sequence was undertaken.

### 4.14 Electrophysiological Recording

ECoG recordings were obtained using the four implant‐integrated electrodes (active site area 1.2×1.2 mm^2^). Recordings were performed in bipolar configuration by pairing electrodes diagonally across the cooling face. Electrodes are separated by approximately 6 mm. Signal conditioning and conversion was performed by hardware on the auxiliary driver unit (RHD2216, Intan Technologies, USA). The internal low‐pass filter was set to 128 Hz and the high‐pass filter was set to 0.5 Hz. Signals were acquired with a 16‐bit resolution at a 256 Hz sample rate. Each signal sample is streamed out to the PC connected to the auxiliary driver as they are sampled. Neural recordings were simultaneously performed using the 16‐channel microelectrode depth probe (D1‐620m‐m10‐0177, NeuroNexus). Electrodes have active site area of 177 µm^2^ and are spaced at a 100 µm pitch. As reference and ground, we used a stainless‐steel wire inserted under the skin at the back of the rat's neck. The probe was connected to a pre‐amplifier (Medusa Bioamp RA16PA, Tucker‐Davis‐Technologies, USA) and then to a data acquisition system (RZ5, Tucker‐Davis‐Technologies, USA) via an optical link. Signals from each channel were sampled at 24.4 kHz rate with 16‐bit resolution. Synchronisation markers were encoded on both (ECoG and LFP) recordings to enable data alignment during analysis. These synchronisation markers are generated by the LabVIEW GUI when the state of the TEC is changed. An Arduino Micro board (Arduino, Italy) was used to generate a 3.3 V pulse for each marker. This signal is routed to an input trigger line on the RZ5 data acquisition system.

### 4.15 Neural Signal Processing

Depth probe LFP and implant ECoG neural recordings were processed offline within MATLAB (r2024b, Mathworks). In a pre‐processing step, a low‐pass filter with a 254 Hz cut‐off frequency was applied before the LFP recordings were down sampled to 508 Hz to speed up compute time. The ECoG recordings were fed through a set of digital band‐stop filters with centre frequencies at 14, 28, and 42 Hz and stopband widths of 2 Hz to remove specific noise generated by the thermocouple amplifier ICs (MAX31855, Analog Devices, USA). Using the event synchronisation markers, signal segments associated with individual cooling events were extracted from both LFP and ECoG recordings. Cooling events that coincided with periods of seizure instability or that were affected by excessive broadband noise were manually identified and excluded from further processing. Cooling events where the desired *T_ref_* could not be reached were not analysed (Table S2).

Comparison of LFP and ECoG recordings in the frequency domain was performed by calculating the power spectral density of 60 s signal slices using the Welch power estimation method with a 10 s Hanning window and 50% window overlap (Figure 4C,D). The instantaneous power of the neural signal was calculated by squaring the sampled signal voltages. The energy in the neural signal was then calculated by applying the ‘trapz’ MATLAB function over a set time window. Normalised signal energy was obtained by dividing the neural signal energy during the final 30 s of cooling by the neural signal energy in the 30 s prior to TEC activation (Figure 4G). Ictal events were extracted from the LFP recordings through a MATLAB script using a threshold to filter for the spikes. LFP data was sliced into 0.5 s windows with 0% overlap. For each window, the maximum ictal spike amplitude was scaled by 0.5 to give the threshold value. Spiking rate and amplitude of each cooling event was averaged within the final 30 s of cooling before being normalised against the 30 s prior to TEC activation. The normalised spiking data was then collated by the *T_ref_* of the cooling event and averaged (Figure 4H,I). Representative spectrograms during cooling events were generated using the ‘spectrogram’ function in MATLAB with a 1.5 s Hanning window and 80% window overlap (Figure 5C). To evaluate the effect of cooling on specific neurological frequency bands, ECoG signals were processed with a set of digital bandpass filters corresponding to the delta (1–4 Hz), theta (4–8 Hz), alpha (8–12 Hz), and beta (12–30 Hz) bands. Signal power was calculated as described above. The normalised signal power was obtained by dividing the instantaneous power by the average power in a 30 s window just before the start of cooling. For visual clarity, a 1 s moving average window was applied (Figure 5D and Figure S11). The normalised signal energy (by frequency band) was calculated as described previously (Figure 5E).

### 4.16 Statistics

Mean data points were generated through the “std” MATLAB function (*w*  =  1) and are displayed with ± 1SD. Statistical significance was calculated using one‐way ANOVA testing (Kruskal Wallis) and Post Hoc processing (Dunn‐Sidak approach). Statistical significance is reached when *p* < 0.05 and is denoted by ^*^. When *p* < 0.001 is reached, it is denoted by ^**^.

### 4.17 Illustrations

Renders and schematics were designed using Blender 5.0 (Blender Foundation) under GNU General Public License or Inkscape (v1.4.2) under GNU General Public License.

## Author Contributions

I.R.M. and J.B. conceived the idea. S.R.M. and T.P. designed and tested the implant. A.C.D.S. performed impedance measurements. J.B. and C.H. developed the seizure model. N.K. and T.P. developed and performed surgical techniques. N.K., T.P, and S.R.M. performed electrophysiological recordings. S.R.M. analysed electrophysiological recordings. S.R.M., I.R.M., T.P. and J.B. wrote the manuscript. All authors revised the manuscript.

## Conflicts of Interest

The authors declare no conflicts of interest.

## Supporting information




**Supporting File**: advs77831‐sup‐0001‐SuppMat.docx.

## Data Availability

The raw experimental data sets and programmes written for this work are available via the OPARA research data repository. https://doi.org/10.25532/OPARA‐1560.
