# Supplementary material for: Printing‐Assisted Integration of Thermal, Fluidic and Electrical Components in Implants for Focal Brain Cooling
Source: Adv Sci (Weinh). 2026 Sep 27:e77831. Online ahead of print. doi: 10.1002/advs.77831 (PMC13616253; doi:10.1002/advs.77831)
Supplement: Supplementary file 1 [file ADVS-9999-e77831-s001.docx]

**Supplementary Materials for: Printing-assisted Integration of Thermal, Fluidic and Electrical Components in Implants for Focal Brain Cooling**

Spencer Ryan Moore^1,2^, Naomi King^3,4^, Aruã Clayton Da Silva^5^, Clare Howarth^3,4^, Jason Berwick^3,4^*, Thomas E. Paterson^4,6,7^* and Ivan R. Minev^1,2^*

^1^Division of Polymer Biomaterials Science, Leibniz Institute for Polymer Research Dresden, Dresden, Germany

^2^Else Kröner Fresenius Center for Digital Health, Medical Faculty Carl Gustav Carus, TUD Dresden University of Technology, Dresden, Germany

^3^Department of Psychology, University of Sheffield, Sheffield, United Kingdom

^4^Neuroscience Institute, University of Sheffield, Sheffield, United Kingdom

^5^Department of Chemical Sciences, SSPC the Research Ireland Centre for Pharmaceuticals, Bernal Institute, University of Limerick, Limerick, Ireland

^6^School of Clinical Dentistry, University of Sheffield, Sheffield, United Kingdom

^7^Insigneo Institute, University of Sheffield, Sheffield, United Kingdom

*corresponding authors:

minev@ipfdd.de

t.paterson@sheffield.ac.uk

j.berwick@sheffield.ac.uk

**Supplementary Figures**

**Figure S1. In silico modelling of TEC heat management solutions.**

**A.** Centre slice view of the in silico model designed in COMSOL (Multiphysics 5.5) where cerebrospinal fluid (CSF) acts as coolant. It consists of 3 material layers: neural tissue, PDMS, and CSF. Thermal properties of the biological tissues were derived from the IT’IS Foundation database [1]. A micro-TEC is embedded within the PDMS layer. TEC dimensions are based on those provided by TEC Microsystems GmbH, Germany for the element embedded in the implant. The TEC semiconductor elements are modelled using the built-in bismuth telluride COMSOL material property. A stainless-steel plate on the TEC cold-side provides the interface with the neural tissue. A finned, stainless-steel heat sink is mounted on the TEC hot-side. The heat sink protrudes into the CSF flow with cylindrical fins in a 6 × 5 grid array pattern. CSF flow rate (0.304 mm s^-1^) was derived from values reported in literature [2, 3]. **B.** Heat map of the system 13 s after activation of the TEC. In the simulation, the TEC is supplied with a constant current of 0.2 A. The temperature of the system is measured at three locations (point probes) indicated with purple markers. **C.** Temperature map (13 s after TEC activation) of the boundary between heat sink and CSF. Flow lines of the CSF are mapped onto the simulation to indicate the CSF flow direction over the heat sink. **D.** Thermal point probe temperatures recorded over the simulation period. Neural tissue in contact with the cold-side steel plate was cooled down to 34.7 °C by the end of the simulation. The TEC hot-side becomes heated to 47.4 °C and the CSF downstream of the heat sink had been heated to 42.5 °C. These temperatures are beyond the biological safety limits of neural tissues.

**Figure S2. Construction of the TEC Assembly.**

**A.** Digitally rendered representation (Blender 5.0, Blender Foundation) of the discrete components package containing the TEC unit, hot-side thermocouple, the cold-side thermocouple, and the stainless-steel heat sink. **B.** A photograph of the finished discrete components package.

**Figure S3. Implant Construction flowchart and design of printing paths.**

**A.** Flow diagram of the production process of the implant**. B.** Neural recording module layers. **C.** Neuromodulation module layers. **D.** Thermal management module layers. All layers – with the exception of layer 3 – are formed using SE1700. Within layer 3 are the four graphite-PDMS electrode paths that are individually printed with material flow discontinuity between them. For B-D, solid black lines denote the current layer (print nozzle) path. Grey lines indicate the pathing of the previously printed layers. Dashed lines indicate where the printhead is still in motion but material extrusion has halted. Discrete symbols denote the start point of the path (green arrow), the end point of the path (red square), and continuous inter-layer transition points (orange arrow). Diagrams designed in Inkscape (v1.4.2).

**Figure S4. Subsystems of the auxiliary driver unit.**

Block diagram of the auxiliary driver unit with its subsystems. Each block is a discrete integrated circuit component. Each block lies within a shaded zone indicating which independently regulated power zone it belongs to. Paths between the blocks illustrate the data and power routing – the light green paths are data links and dark green paths are the power buses. Pneumatic feeds of the piezoelectric air pump and air valve systems are indicated in grey. The arrows represent the external connections from the auxiliary driver. Diagram designed in Inkscape (v1.4.2)

**Figure S5. Implementation of the microfluidic thermal management subsystem.**

**A.** System diagram of the thermal management hardware. A piezoelectric air pump (UXPB5401200A, The Lee Company, USA) was used to pressurise coolant reservoir A – driving the coolant fluid flow. The coolant (deionised water) is routed through the silicone tubing to the thermal management microfluidic loop on the implant. Coolant then flows out into coolant reservoir B. Larger silicone tubing (I.D. = 3 mm, O.D. = 5 mm) was used for the majority of the flow path. A 3D printed adapter was used to interface the larger diameter silicone tubing to the small tubing of the implant (I.D. = 0.4 mm, O.D. = 1.0 mm). The direction of coolant flow is dictated by a pair of air valves (18801105, Series 188, Emerson, USA) that work in tandem to control the application of air pressure to the reservoirs. A flow sensor (SLF3S-1300B, Sensirion, Switzerland) was placed in the coolant flow path to monitor system performance. The two coolant reservoirs were constructed from 20 ml syringes. 3D printed tubing mounts were glued to the plunger opening. PTFE filters (100 µm pore size, PF100, ADVANTEC, Japan) were installed in the syringes to prevent water ingress into the pump/valve section. Diagram designed in Inkscape (v1.4.2). **B.** Photograph of the thermal management hardware shield populated with the electronic components required to power and control the pump and valves. Connection to the main auxiliary driver board was achieved through a 10-pin picoblade connector (Molex, USA). **C.** Photograph of the full thermal management hardware shield setup with the piezoelectric air pump SPM-041 module and valves mounted.

**Figure S6. Estimation of the volume affected by cooling from the implant.**

**A.** Thermal image of an implant delivering cooling to a block of heated Agar gel (see methods). The image was taken 60s into a cooling event (*T_ref_* = 18 °C). The boundary profile shown by a broken line is an isotherm along which a -2 °C change of temperature in the Agar gel is detected. The thermal resolution of the camera is approximately 2 °C. In other words, the Agar gel within the boundary experiences TEC induced cooling, while outside, the cooling effect is undetectable. **B.** Rendering (Blender 5.0, Blender Foundation) of the estimated volume affected by the TEC using the boundary profile in A. The dimensions of the volume affected by cooling are given in x, y, and z axis. This produced an estimated cooled volume of approximately 38 mm^3^ after 60 s of TEC action to *T_ref_* = 18 °C.

**Figure S7. Simulated cooling profile within non-perfused and perfused neural tissue.**

**A.** Simplified implant cooling model on non-perfused neural tissue and the resultant cooling profile after 35s of elapsed simulation time. Simulated using COMSOL Multiphysics 5.5. Thermal properties of the neural tissue were derived from the IT’IS Foundation database [1]. The simplified implant model is set to 20 °C after 5 s of simulation time had elapsed. The profile mirrors the captured in vitro thermal images (Figure 3I) which was expected due to the agar gel model and the neural tissue having similar thermal properties. **B.** Simplified implant cooling model on perfused neural tissue and the resultant cooling profile after 35 s of elapsed simulation time. Perfusion and metabolic heat generation within the tissue is modelled using the Penne’s bioheat equation. Perfusion within the simulated tissue produces a thermal buffering against the cooling action of the simplified implant. The resultant thermal profile indicates a shallower penetration of the cooling effect into the tissue and a restriction of cooling to directly beneath the interface between the tissue and cooling source.

**Figure S8. Surgical procedure and implant placement.**

**A.** Instrument setup diagram for the in vivo experimental sessions. All recording was performed within a faraday cage. Data links for the NeuroNexus depth probe and implant were passed through opto-isolators to electrically isolate the devices. Both systems were powered by dedicated battery packs during experimentation. Diagram designed in Inkscape (v1.4.2). **B.** An optical micrograph of a representative cranial window before placement of the implant. The green light source highlights arterial and venous blood supplies in the cranial window to aid in implant placement. **C.** Mounting of the implant and NeuroNexus depth probe in the cranial window. A 3D printed structure and cotton swab were used to maintain implant position on the dura mater. **D.** General view of the full experimental setup inside the faraday cage. All instruments and metal objects are connected to a single star point ground on the faraday cage platform to prevent ground loops.

**Figure S9. Effects of focal brain cooling on LFP energy (depth probe recordings) in individual rats.**

Normalised signal energy for each *T_ref_* and each experimental subject. (**A – I**) Rat 1-9 respectively. For clarity, a selection of electrodes is shown. Small symbols indicate the normalized signal energy for each cooling event **(Supplementary Table 2)**. In rats where fewer than three cooling events were analysed (e.g., *T_ref_*  = 15 °C in A), no analysis of statistical significance is performed. The animal average (large symbols) is obtained by averaging individual cooling events. Error bars represent one standard deviation. Statistical significance is indicated with * when *p* < 0.05 and ** when *p* < 0.01.

**Figure S10. Testing for switching artifacts during cooling event.**

**A.** In vitro setup for testing the impact of the thermal modulation sequence during a cooling event on the electrode recordings. An implant is embedded within an electrically conductive Agar-PBS gel. The auxiliary driver is connected to the implant for driving the system and data is streamed to a linked PC. During testing, cooling events were conducted (*T_ref_* = 20 °C) whilst the electrode recordings were monitored for artifacts. Diagram designed in Inkscape (1.4.2) **B.** A representative implant ECoG recording whilst a cooling event is undertaken. Artifacts were not observed across the cooling event. The activation periods of the neuromodulation and thermal management hardware are highlighted on the graph.

**Figure S11. Effect of *T_ref_* on seizure suppression across neurological frequency bands.**

Normalised ECoG signal power (whole cohort averaged) separated by *T_ref_*: **A.** 25 °C, **B.** 20 °C, **C.** 18 °C, **D.** 15 °C, and **E.** sham cooling. For each *T_ref_* signals are filtered into neural frequency bands: **i.** Delta band, **ii.** Theta band, **iii.** Alpha band, **iv.** Beta band. Each averaged signal power trace has ± 1 SD represented by a shaded area about the time-series line.

**Supplementary Tables**

|  | **Post anaesthesia** | **Post craniotomy** | **Post cooling events** |
| --- | --- | --- | --- |
| pH | 7.384 ± 0.006 | 7.371 ± 0.018 | 7.369 ± 0.008 |
| pCO_2_ [mmHg] | 28.9 ± 1.7 | 34.0 ± 6.2 | 45.2 ± 1.6 |
| p0_2_ [mmHg] | 83 ± 4 | 92 ± 5 | 65 ± 7 |
| s0_2_ [%] | 96 ± 1 | 97 ± 1 | 91 ± 3 |

**Table S1. Intraoperative animal monitoring.**

Blood gas and pH measurements from *n* = 3 rats collected at three time points during the experiment; immediately after induction of anaesthesia, following completion of the craniotomy, and at the conclusion of the experiment after implant removal. Values are presented as mean ± SD.

| **Experimental Animal** | **Temperature Reference [*T_ref_*]** | | | | | | | | | | | | | |
| --- | --- | --- | --- | --- | --- | --- | --- | --- | --- | --- | --- | --- | --- | --- |
|  | **25 °C** | |  | **20 °C** | |  | **18 °C** | |  | **15 °C** | |  | **Sham** | |
|  | **Depth Probe** | **Implant** |  | **Depth Probe** | **Implant** |  | **Depth Probe** | **Implant** |  | **Depth Probe** | **Implant** |  | **Depth Probe** | **Implant** |
| Rat 1 | 4 | 0 |  | 4 | 7 |  | 3 | 3 |  | 1 | 2 |  | 7 | 7 |
| Rat 2 | 0 | 0 |  | 7 | 3 |  | 8 | 2 |  | 6 | 2 |  | 16 | 16 |
| Rat 3 | 4 | 6 |  | 6 | 8 |  | 3 | 4 |  | 8 | 8 |  | 16 | 16 |
| Rat 4 | 4 | 1 |  | 5 | 2 |  | 3 | 1 |  | 3 | 1 |  | 14 | 14 |
| Rat 5 | 4 | 0 |  | 6 | 0 |  | 9 | 2 |  | 0 | 0 |  | 16 | 16 |
| Rat 6 | 3 | 3 |  | 4 | 4 |  | 5 | 0 |  | 2 | 0 |  | 13 | 13 |
| Rat 7 | 5 | 1 |  | 7 | 4 |  | 6 | 6 |  | 0 | 0 |  | 14 | 14 |
| Rat 8 | 4 | 2 |  | 7 | 6 |  | 5 | 4 |  | 2 | 0 |  | 6 | 6 |
| Rat 9 | 0 | 0 |  | 4 | 0 |  | 2 | 0 |  | 0 | 0 |  | 7 | 0 |
| Total Events | 28 | 13 |  | 50 | 34 |  | 44 | 22 |  | 22 | 13 |  | 109 | 102 |

**Table S2. Cumulative number of cooling events for each rat in the experimental animal cohort.**

A log of cooling events after the exclusion criteria (section **Neural Signal Processing**) was applied. Although the LFP and ECoG recordings were performed in parallel, excessive broadband noise was occasionally observed with one of the systems neural recordings but not the other. Cooling events undertaken during periods of observed excessive noise within LFP or ECoG recordings were manually excluded from that recording modalities pool of cooling events for further processing.

| **Material** | **BioCAD** | | | |  | **3D Discovery Printer & HMI** | | | | |
| --- | --- | --- | --- | --- | --- | --- | --- | --- | --- | --- |
|  | **Feed Rate [mm s^-1^]** | **Layer**  **Thickness [mm]** | **Printhead** | **Extrusion**  **Delay [1/16 s]** |  | **Nozzle**  **Size [mm]** | **Feed Pressure**  **[MPa]** | **Syringe**  **Volume** | **Plunger**  **Velocity**  **[mm s^-1^]** | **Volume Flow**  **Rate [μL s^-1^]** |
| SE1700 [Base] | 5.0 | 0.210 | PSD | 300 |  | 0.234 | 0.340 | 3 cc | n/a | n/a |
| SE1700 [90°] | 5.5 | 0.228 | PSD | 300 |  | 0.234 | 0.340 | 3 cc | n/a | n/a |
| SE1700 [45°] | 5.5 | 0.162 | PSD | 300 |  | 0.234 | 0.340 | 3 cc | n/a | n/a |
| Graphite-PDMS [1:1] | 4.0 | 0.220 | VSD | 200 |  | 0.335 | n/a | 2.5 mL | 0.014 | 1.1 |

**Table S3. RegenHU 3D Discovery bioprinter material printing profiles.**

Printer material printing profiles enable fine control over the pneumatic strand dispensing and volumetric strand dispensing printheads. **Feed rate** is the translational movement velocity of the printhead in mm s^-1^. **Layer thickness** is the z-axis offset relative to the previous layers z-axis height in mm. The **Printhead** setting indicates which deposition method – and therefore printhead - is used. **Extrusion delay** is a programmed pause during an extrusion start event, to account for previous material retraction or establishing a stable syringe air pressure, and an extrusion stop event, to allow for material retraction or to prevent possible material oozing. The total time of these delays is calculated by the printer by multiplying the programmed value by 1/16 s. **Nozzle size** is the inner diameter of the mounted nozzle to be extruded through. **Feed pressure** is a setting specific to the pneumatic strand dispenser (PSD) and is the magnitude of the air pressure in kPa to be applied to the mounted syringe. For the PSD printheads, the **syringe volume** only determines which mounting adapter is required for the printhead setup. With the volumetric strand dispenser (VSD) settings, the syringe volume value has to be inputted into the 3D Discovery HMI software, along with the **plunger velocity,** to system to calculate the desired **volume flow rate**.

Multiple SE1700 material settings were developed for the optimisation of section printing. The annotation [Base] has a lower feed rate and layer thickness resulting in an increase of SE1700 deposited during printing. For the neural recording module, this is crucial for reliably insulating the printed electrodes. The annotations [90°] and [45°] with the select material titles indicate the altitude angles of the current print layer compared to the previous layer. An altitude angle of [90°] indicates that the printed filaments are vertically stacked whilst the [45°] material results in an offset (overhang) that is crucial for constructing the ‘roof’ of microfluidic channels.

| **Implant Module** | **Layer Number** | **Assigned Material Settings** |
| --- | --- | --- |
| Neural Recording | Layer 1 | SE1700 Base |
|  | Layer 2 | SE1700 Base |
|  | Layer 3 | Graphite-PDMS [1:1] |
| Neuromodulation | Layer 4 | SE1700 Base |
|  | Layer 5 | SE1700 Base |
|  | Layer 6 | SE1700 Base |
| Thermal Management | Layer 7 | SE1700 [90°] |
|  | Layer 8 | SE1700 [90°] |
|  | Layer 9 | SE1700 [90°] |
|  | Layer 10 | SE1700 [45°] |
|  | Layer 11 | SE1700 [45°] |
|  | Layer 12 | SE1700 [45°] |
|  | Layer 13 | SE1700 [45°] |

**Table S4. Assignment of the material printing profiles to the individual implant layers.**

Assignment of the material and printing parameters to the individual implant layers is indicated. These settings are applied to the layer pathing within the BioCAD software during the implant G-code generation process. Three separate material profiles have been optimised around the structural requirements of each implant module and path purpose.

**Supplementary References**

1	Baumgartner, C. *et al.* IT’IS Database for thermal and electromagnetic parameters of biological tissues. (2025). https://doi.org/10.13099/VIP21000-05-0

2	Lee, J. H. *et al.* CSF Flow Quantification of the Cerebral Aqueduct in Normal Volunteers Using Phase Contrast Cine MR Imaging. *Korean J Radiol* **5**, 81-86 (2004). https://doi.org/10.3348/kjr.2004.5.2.81

3	Yousef, M. I., Abd El Mageed, A. E., Yassin, A. E. N. & Shaaban, M. H. Use of cerebrospinal fluid flow rates measured by phase-contrast MR to differentiate normal pressure hydrocephalus from involutional brain changes. *The Egyptian Journal of Radiology and Nuclear Medicine* **47**, 999-1008 (2016). https://doi.org/https://doi.org/10.1016/j.ejrnm.2016.04.001

4	Zhang, M. *et al.* Experimental Determination of Thermal Conductivity of Water−Agar Gel at Different Concentrations and Temperatures. *J. Chem. Eng. Data* **56**, 859–864 (2011). https://doi.org/10.1021/je100570h
